# Coronary Microvascular Dysfunction and Major Adverse Cardiovascular Events in Patients With Ischemia and Non-obstructive Coronary Arteries: A Systematic Review

**DOI:** 10.7759/cureus.115077

**Published:** 2026-08-24

**Authors:** Osama Waleed Abdallah, Fatma Attia, Ghada M Al-Saleh, Ahmad Alaqabawi, Muath A Mustafa, Ahmad Difallah Al Darawsheh, Nermeen Abuhalaweh, Mohannad O Allasasmeh, Zaid A Al-Samarat, Lujain Somrain, Raghad Abuali

**Affiliations:** 1 Internal Medicine, Prince Hamza Hospital, Amman, JOR; 2 Faculty of Medicine, University of Jordan, Amman, JOR; 3 Internal Medicine, Mutah University, Amman, JOR; 4 Internal Medicine, University of Jordan, Amman, JOR

**Keywords:** coronary flow reserve, coronary microvascular dysfunction, ischemia with non-obstructive coronary arteries, major adverse cardiovascular events, microvascular angina, prognosis

## Abstract

Coronary microvascular dysfunction (CMD) is a major pathophysiological substrate of ischemia with non-obstructive coronary arteries (INOCA), but available prognostic evidence remains heterogeneous in diagnostic methods, CMD definitions, outcome definitions, and reported effect measures. We conducted a systematic review with narrative synthesis of cohort studies examining the association between objectively verified CMD and major adverse cardiovascular events (MACE) in adults with INOCA. MEDLINE/PubMed, Embase, Cochrane CENTRAL, Scopus, and Web of Science were searched from inception to June 2026. Eight cohort studies representing eight unique countries met eligibility criteria and were included in the narrative synthesis. Two independent cohorts reported adjusted dichotomous hazard ratios for a CMD-versus-no-CMD contrast: HR 2.97, 95% CI 1.39-6.34 and HR 3.12, 95% CI 1.22-7.97, respectively. The largest and highest-quality included study (N=1,681, NOS 9/9) reported an adjusted hazard ratio for continuous coronary flow velocity reserve and was summarized narratively. The remaining studies reported non-comparable effect measures, exposure scales, adjustment levels, or overlapping cohorts. Because of heterogeneity in CMD definitions, diagnostic modalities, exposure scales, and effect-measure types, no meta-analysis was performed. Available observational evidence suggests that objectively identified CMD may be associated with increased MACE risk in patients with INOCA. Larger prospective studies using standardized CMD definitions, harmonized MACE outcomes, adequate follow-up, and consistently adjusted time-to-event analyses are needed.

## Introduction and background

Ischemia with non-obstructive coronary arteries (INOCA) is increasingly recognized as a common and clinically consequential presentation of ischemic heart disease. A substantial proportion of patients evaluated for stable angina have no flow-limiting epicardial stenosis, yet many experience persistent symptoms, recurrent health-care use, impaired functional status, and reduced quality of life [1-3]. Long-term evidence has also established that INOCA is not a benign diagnosis and is associated with excess cardiovascular morbidity compared with populations without ischemic heart disease [1,4].

Coronary microvascular dysfunction (CMD) represents a major mechanistic substrate of INOCA. Structural remodeling, endothelial dysfunction, impaired vasodilator reserve, abnormal vasomotor regulation, and increased microvascular resistance may restrict myocardial blood-flow augmentation during stress and contribute to recurrent ischemia [2,5,6]. CMD can be assessed invasively using coronary flow reserve (CFR), the index of microvascular resistance (IMR), hyperemic microvascular resistance, or angiography-derived indices, and non-invasively using echocardiographic coronary flow velocity reserve, positron-emission tomography, cardiovascular magnetic resonance, or single-photon-emission computed tomography [2,4,6].

Standardized diagnostic criteria have improved conceptual clarity, but substantial variability remains in testing modality, threshold selection, population characteristics, and outcome definition [4,6]. This variability is important because impaired microvascular function has been associated not only with recurrent angina but also with myocardial injury, heart failure, and adverse cardiovascular events [3,7,8].

Contemporary aggregate-data syntheses have reported that CMD is associated with increased cardiovascular risk across broad populations, but available reviews have combined heterogeneous populations, diagnostic modalities, CMD thresholds, and effect-measure types [3,9]. Several recent reviews have also emphasized that prognosis in INOCA differs according to vasomotor endotype, diagnostic modality, and whether coronary flow reserve and microvascular resistance are concordantly or discordantly abnormal. Therefore, a focused systematic review is needed to map the prognostic evidence in INOCA, clarify which effect estimates are directly comparable, and identify why reliable quantitative pooling remains limited.

The objective of this systematic review was to describe the association between objectively diagnosed CMD and MACE in patients with INOCA, summarize individual prognostic estimates narratively, and identify methodological barriers to future quantitative synthesis.

## Review

Materials and methods

*Protocol and Registration* This review was conducted and reported in accordance with the PRISMA 2020 statement [10]. The protocol was registered prospectively on the Open Science Framework before data extraction [11].

Eligibility Criteria

Prospective and retrospective cohort studies were eligible if they enrolled adults with INOCA, formally assessed CMD, and reported MACE according to CMD status with an extractable effect estimate. Case reports, reviews, editorials, conference abstracts, non-cohort designs, studies without formal CMD assessment, and reports without extractable outcome data were excluded.

The review was designed to comprehensively map prognostic evidence on the CMD-MACE association in INOCA across all reported effect-measure types. Because the included studies used heterogeneous exposure scales and effect measures, estimates were not pooled when they were reported as continuous effects, odds ratios, incidence-rate ratios, unadjusted estimates, or when cohorts overlapped. All eligible studies were retained in the narrative synthesis, and the reason each study was summarized descriptively or narratively is shown in the effect-measure eligibility table.

Information Sources and Search Strategy

MEDLINE/PubMed, Embase, Cochrane CENTRAL, Scopus, and Web of Science were searched from inception through June 2026. Reference lists of included studies and relevant reviews were also screened.

The master search strategy used the following Boolean combination: ("coronary microvascular dysfunction" OR "microvascular angina" OR "coronary flow reserve" OR "coronary flow velocity reserve" OR "myocardial flow reserve" OR "index of microvascular resistance" OR CMD OR CFR OR CFVR OR MFR OR IMR) AND (INOCA OR "ischemia with non-obstructive coronary arteries" OR "ischaemia with non-obstructive coronary arteries" OR "non-obstructive coronary artery disease" OR "no obstructive coronary artery disease") AND (prognosis OR prognostic OR outcome OR outcomes OR mortality OR death OR "major adverse cardiovascular events" OR MACE OR "cardiovascular events" OR "myocardial infarction" OR "heart failure" OR follow-up). This strategy was adapted for each database using database-specific controlled vocabulary, field tags, and syntax.

Study Selection and Data Extraction

Two reviewers independently screened titles and abstracts, assessed full texts, and extracted study characteristics, population data, CMD definitions, outcome definitions, adjusted effect estimates, and follow-up information using a piloted form. Disagreements were resolved by consensus, with third-reviewer adjudication when required. Every candidate effect estimate reported by each included study was catalogued in an effect-measure eligibility audit that recorded the effect type, exposure contrast, adjustment status, exposure scale, and reason each estimate was summarized descriptively or narratively. Potentially overlapping source cohorts were examined before synthesis, and overlapping reports were not treated as independent evidence.

Risk of Bias Assessment

Two reviewers independently assessed methodological quality using the Newcastle-Ottawa Scale (NOS) for cohort studies [12]. The assessment covered the nine NOS items across the selection, comparability, and outcome domains: cohort representativeness, selection of the non-exposed cohort, exposure ascertainment, demonstration that the outcome was absent at baseline, adjustment for main confounders, adjustment for additional confounders, outcome assessment, adequacy of follow-up length, and completeness of follow-up. Domain-level judgments are presented in a study-level traffic-light plot and a domain-summary plot.

Statistical Analysis

The primary objective was to summarize the prognostic evidence on the association between objectively defined CMD and MACE in INOCA according to effect-measure type, exposure scale, CMD modality, and adjustment status. Individual effect estimates were tabulated and compared descriptively. Because the included studies differed in CMD definitions, diagnostic modalities, exposure scales, and effect-measure types, no meta-analysis was performed. Two independent cohorts reported adjusted dichotomous hazard ratios for a CMD-versus-no-CMD contrast and were summarized descriptively. Studies reporting continuous estimates, odds ratios, incidence-rate ratios, unadjusted estimates, alternative CMD definitions, or overlapping cohorts were summarized narratively according to the effect-measure eligibility audit shown in Table 2. Heterogeneity statistics, funnel-plot asymmetry testing, influence analyses, and meta-regression were not performed because no pooled model was fitted.

Results

Study Selection

**Figure 1:**
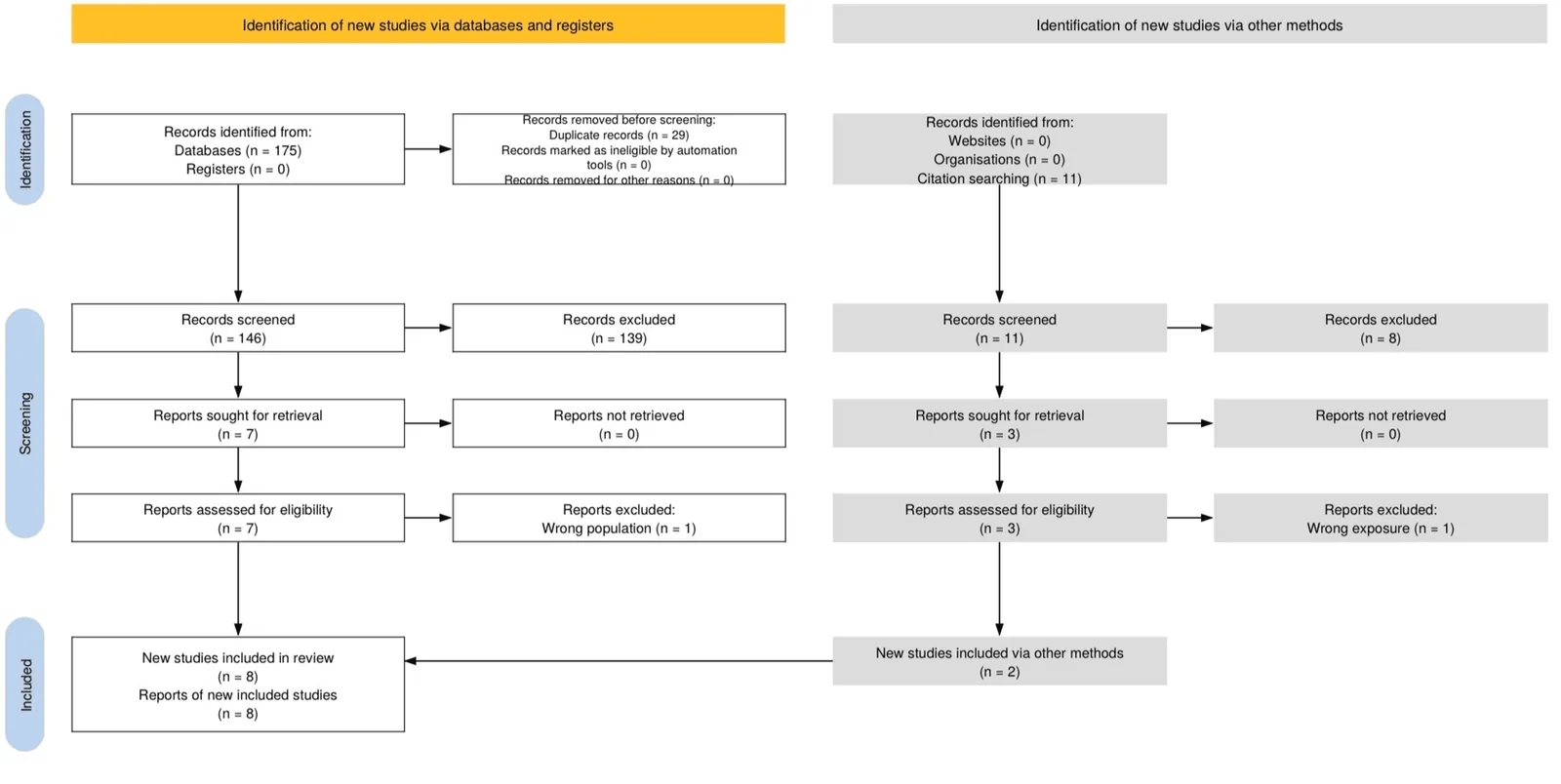
PRISMA 2020 flow diagram of study identification, screening, eligibility, and inclusion

The search identified 186 records in total, including 175 records from database searching and 11 records from citation searching. No records were identified from registers, websites, or organizations. After removal of 29 duplicate database records, 146 database records underwent title and abstract screening, of which 139 were excluded. Seven database reports were sought and retrieved, and all seven were assessed for eligibility; one was excluded because of an ineligible population, leaving six studies included from database searching. Citation searching identified 11 additional records; all 11 were screened, eight were excluded, and three full-text reports were sought, retrieved, and assessed. One citation-searching report was excluded because of an ineligible exposure, leaving two studies included from citation searching. Overall, eight cohort studies, reported in eight reports, were included in the systematic review (Figure 1).

Study Characteristics

The eight included cohort studies were published between 2021 and 2026, representing eight countries [5,6,13-18]. Five studies used invasive physiological assessment, and three used non-invasive assessment. Study characteristics are summarized in Table 1.

**Table 1:** Characteristics of the included cohort studies Characteristics of the eight included cohort studies, including design, analytic population, CMD assessment modality and definition, and Newcastle-Ottawa Scale score. Mohammed et al. (2023) and Liu et al. (2023) report the same 151-patient cohort. Abbreviations: caIMR, coronary angiography-derived index of microvascular resistance; CFR, coronary flow reserve; CFVR, coronary flow velocity reserve; CMD, coronary microvascular dysfunction; CZT-SPECT, cadmium-zinc-telluride single-photon-emission computed tomography; HMR, hyperemic microvascular resistance; IMR, index of microvascular resistance; MFR, myocardial flow reserve; NOS, Newcastle-Ottawa Scale; PET, positron-emission tomography.

Study [ref]	Country/setting	Design	N	CMD assessment modality	CMD threshold/definition	NOS
Lee et al., 2022 [5]	International: Korea, Netherlands, Japan, Spain, Denmark, Italy, USA	Prospective multicenter registry	287	Invasive Doppler CFR	CFR ≤2.5	8/9
Schröder et al., 2021 [6]	Denmark	Prospective cohort	1,681	Non-invasive echocardiographic CFVR	CFVR <2.25; continuous CFVR also modeled	9/9
Toya et al., 2021 [13]	USA	Observational cohort	610	Invasive CFR and HMR	CFR <2.5 and/or HMR >2.0	7/9
Zampella et al., 2023 [14]	Italy	Retrospective cohort	311	Non-invasive 82Rb PET MFR	MFR <2	8/9
Rumiz et al., 2026 [15]	Spain	Prospective multicenter observational	308	Invasive CFR/IMR with acetylcholine testing	CFR <2.5 and/or IMR ≥25	7/9
Mohammed et al., 2023 [16]	China	Retrospective observational	151	Invasive caIMR	caIMR ≥25	6/9
Liu et al., 2023 [17]	China	Retrospective observational	151	Invasive caIMR	caIMR ≥25	6/9
Li et al., 2023 [18]	China	Retrospective observational	100	Non-invasive CZT-SPECT MFR	MFR <2.0	4/9

The composition of the narrative synthesis followed the prespecified effect-measure eligibility framework. All eight studies were retained in the systematic review and narrative synthesis. Two independent cohorts reported adjusted dichotomous hazard ratios for a CMD-versus-no-CMD contrast: Lee et al. (2022) and Liu et al. (2023) [5,17]. Schröder et al. (2021) was the largest and highest-quality included cohort (N=1,681, NOS 9/9) and reported an adjusted hazard ratio for continuous coronary flow velocity reserve [6]. Although this estimate was not directly comparable with dichotomous CMD-versus-no-CMD hazard ratios, Schröder et al. remains a central component of the evidence base and was summarized narratively. The remaining studies reported effect measures or exposure scales that were not suitable for direct quantitative comparison, including odds ratios, incidence-rate ratios, unadjusted estimates, alternative CMD definitions, or overlapping cohorts [13-18].

Risk of Bias

**Figure 2:**
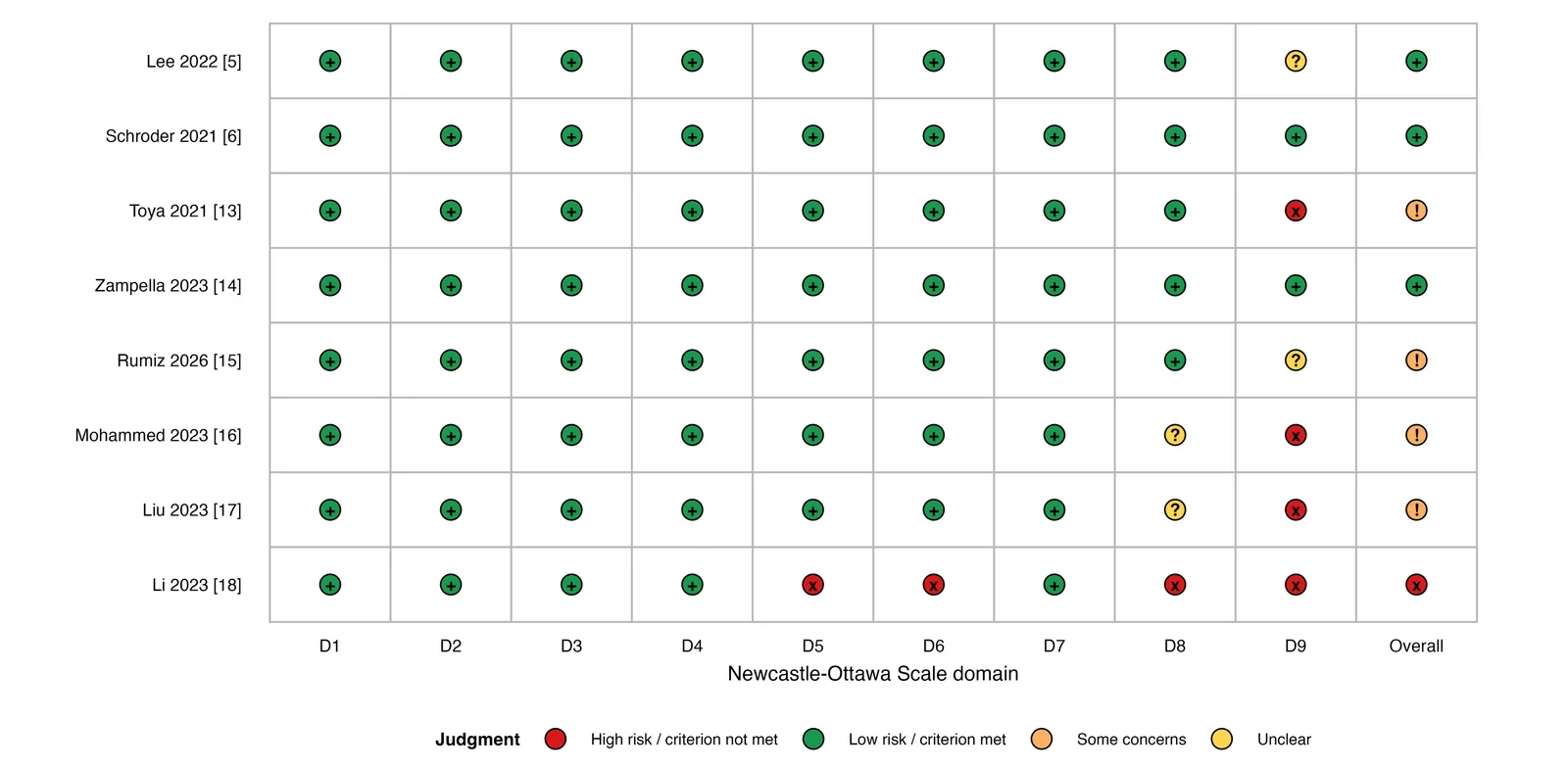
Study-level Newcastle-Ottawa Scale risk-of-bias assessment Traffic-light plot showing the methodological quality of each of the eight included cohort studies across the nine Newcastle-Ottawa Scale items, with each study identified by name and citation number: Lee et al. (2022) [5], Schröder et al. (2021) [6], Toya et al. (2021) [13], Zampella et al. (2023) [14], Rumiz et al. (2026) [15], Mohammed et al. (2023) [16], Liu et al. (2023) [17], and Li et al. (2023) [18]. Domains D1 to D9 are cohort representativeness, selection of the non-exposed cohort, exposure ascertainment, demonstration that the outcome was absent at baseline, adjustment for main confounders, adjustment for additional confounders, outcome assessment, adequacy of follow-up length, and completeness of follow-up. Assessment followed the Newcastle-Ottawa Scale for cohort studies [12].

**Figure 3:**
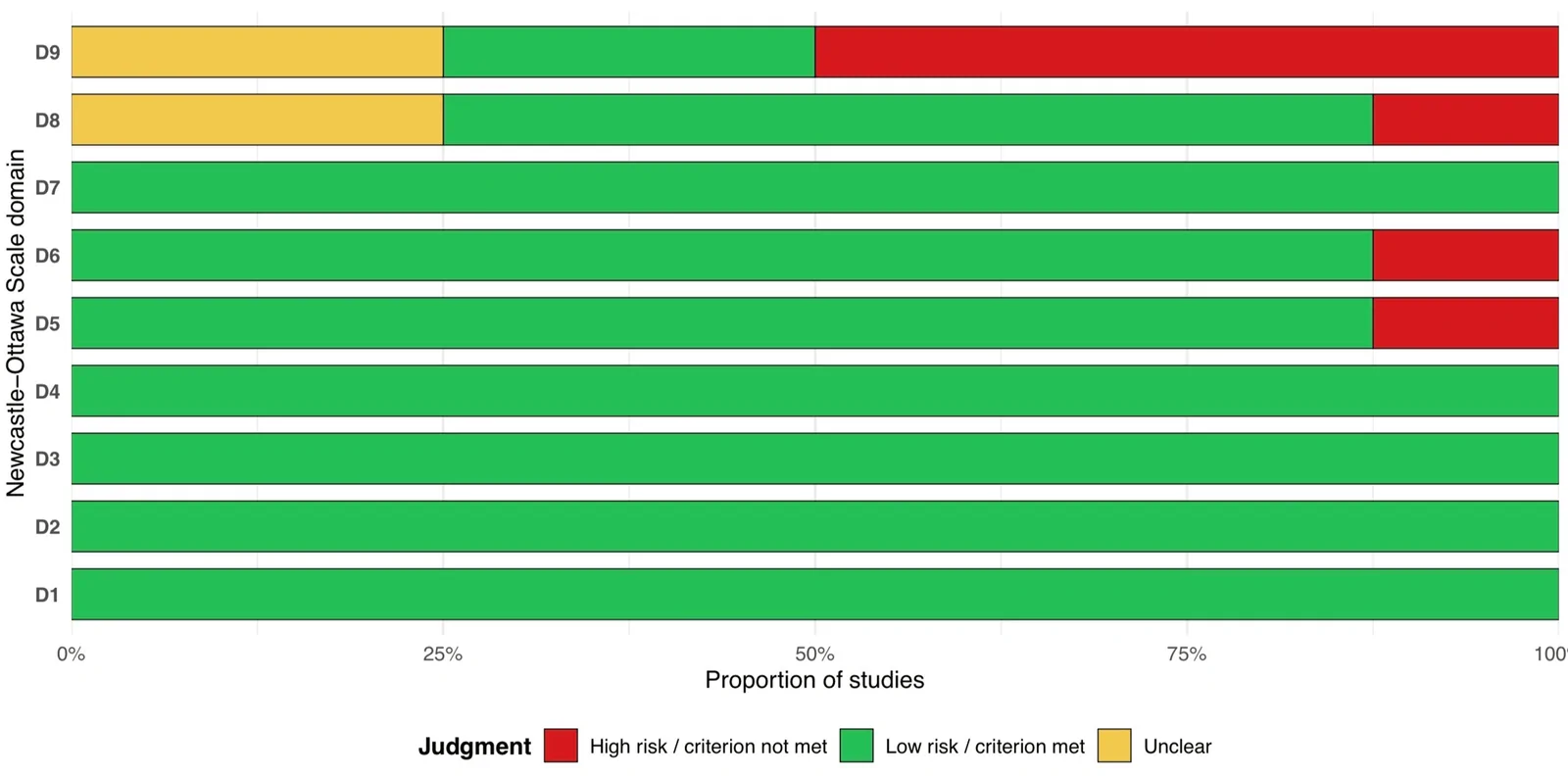
Distribution of Newcastle-Ottawa Scale judgments across quality domains Summary plot showing the proportion of the eight included cohort studies assigned to each risk-of-bias category within each of the nine Newcastle-Ottawa Scale domains and overall [5,6,13-18]. Assessment followed the Newcastle-Ottawa Scale for cohort studies [12].

Methodological quality varied across studies [5,6,13-18]. NOS scores ranged from 4/9 to 9/9. Schröder et al. achieved the highest score (9/9), with complete registry-based follow-up and no patients lost [6]. Lee et al. (2022) and Zampella et al. (2023) each scored 8/9 [5,14]. Toya et al. (2021) and Rumiz et al. (2026) each scored 7/9, and Mohammed et al. (2023) and Liu et al. (2023) each scored 6/9 [13,15-17]. Li et al. (2023) received the lowest score (4/9), mainly because only unadjusted estimates were available for the INOCA subgroup and follow-up was short and incomplete [18]. At the study level, three studies were judged at overall low risk of bias, four raised some concerns, and one was judged at high risk. Figure 2 presents the study-level Newcastle-Ottawa Scale assessment, and Figure 3 summarizes the distribution of judgments across the nine NOS domains.

Individual Study Effect Estimates and Narrative Synthesis

Eight included studies reported prognostic estimates in varying formats. Two independent cohorts reported adjusted dichotomous hazard ratios for the CMD-versus-no-CMD contrast: Lee et al. (HR 2.97, 95% CI 1.39-6.34) and Liu et al. (HR 3.12, 95% CI 1.22-7.97) [5,17]. These estimates were summarized descriptively but were not pooled.

Schröder et al., the largest and highest-quality included cohort (N=1,681, NOS 9/9), reported an adjusted hazard ratio of 1.05 (95% CI 1.01-1.09) per 0.1-unit decrease in coronary flow velocity reserve [6]. Although this estimate was reported on a continuous scale rather than as a dichotomous CMD-versus-no-CMD contrast, it supports the prognostic relevance of impaired coronary flow velocity reserve and remains a central component of the evidence base.

The remaining studies were summarized narratively because they reported continuous estimates, odds ratios, incidence-rate ratios, unadjusted estimates, alternative CMD definitions, or overlapping cohorts. Zampella et al. reported an adjusted HR of 4.47 (95% CI 1.43-13.94) for reduced myocardial flow reserve assessed using 82Rb positron-emission tomography, an exposure definition that was not equivalent to the CMD contrasts used in the two dichotomous HR studies [14]. Toya et al. reported an adjusted OR of 1.55 (95% CI 1.07-2.25), and Rumiz et al. reported an adjusted IRR of 3.24 (95% CI 1.12-9.05); these measures were not directly interchangeable with time-to-event hazard ratios [13,15]. Li et al. reported an unadjusted HR of 4.36 (95% CI 1.15-16.58) for the INOCA subgroup and therefore contributed lower-certainty narrative evidence [18]. Mohammed et al. and Liu et al. originated from the same 151-patient cohort, so only Liu et al. was retained in the descriptive comparison to prevent double counting [16,17].

**Table 2:** Effect-Measure Eligibility and Narrative Synthesis Approach Abbreviations: caIMR, coronary angiography-derived index of microvascular resistance; CFR, coronary flow reserve; CFVR, coronary flow velocity reserve; CMD, coronary microvascular dysfunction; CZT-SPECT, cadmium-zinc-telluride single-photon emission computed tomography; HMR, hyperemic microvascular resistance; HR, hazard ratio; IRR, incidence-rate ratio; MFR, myocardial flow reserve; NOS, Newcastle-Ottawa Scale; OR, odds ratio; PET, positron-emission tomography.

Study	Reported estimate	Adjustment	Direct quantitative comparability	Narrative synthesis approach
Lee et al., 2022 [5]	HR 2.97, 95% CI 1.39-6.34, for CFR ≤2.5 versus no CMD	Adjusted	Comparable with Liu et al. by effect-measure type	Descriptively summarized; not pooled.
Schröder et al., 2021 [6]	Adjusted HR 1.05, 95% CI 1.01-1.09, per 0.1-unit CFVR decrease; unadjusted dichotomous HR 1.94, 95% CI 1.29-2.91	Adjusted for continuous estimate; dichotomous estimate unadjusted	Not directly comparable with dichotomous adjusted HRs	Central narrative evidence because it was the largest and highest-quality cohort (NOS 9/9).
Toya et al., 2021 [13]	Adjusted OR 1.55, 95% CI 1.07-2.25, for CFR/HMR-defined CMD	Adjusted	Not directly comparable with time-to-event HRs	Summarized narratively because odds ratios are not directly interchangeable with hazard ratios.
Zampella et al., 2023 [14]	Adjusted HR 4.47, 95% CI 1.43-13.94, for PET MFR <2	Adjusted	Not directly comparable with CFR/caIMR-defined CMD contrasts	Summarized narratively because the exposure definition and modality differed.
Rumiz et al., 2026 [15]	Adjusted IRR 3.24, 95% CI 1.12-9.05, for invasive CFR/IMR-defined microvascular dysfunction	Adjusted	Not directly comparable with HRs	Summarized narratively because incidence-rate ratios are not directly interchangeable with hazard ratios.
Mohammed et al., 2023 [16]	Adjusted HR 2.73, 95% CI 1.16-6.40, for caIMR ≥25	Adjusted	Overlaps with Liu et al.	Summarized narratively only as overlapping evidence from the same 151-patient cohort.
Liu et al., 2023 [17]	Adjusted HR 3.12, 95% CI 1.22-7.97, for caIMR ≥25 versus no CMD	Adjusted	Comparable with Lee et al. by effect-measure type	Descriptively summarized; retained to avoid double counting.
Li et al., 2023 [18]	Unadjusted HR 4.36, 95% CI 1.15-16.58, for CZT-SPECT MFR <2.0 in the INOCA subgroup	Unadjusted	Not directly comparable with adjusted HRs	Summarized narratively because the INOCA subgroup estimate was unadjusted and the study had lower methodological quality.

Discussion

This systematic review included eight cohort studies examining the association between CMD and MACE in patients with INOCA. Two independent cohorts reported adjusted dichotomous hazard ratios for a CMD-versus-no-CMD comparison, with estimates close to 3, while Schröder et al., the largest and highest-quality cohort, reported an adjusted continuous estimate for coronary flow velocity reserve [5,6,17]. The evidence supports the prognostic relevance of impaired coronary microvascular function, but differences in CMD definitions, diagnostic methods, exposure scales, effect measures, and adjustment strategies prevented reliable quantitative pooling.

These findings are consistent with the view that INOCA is not a benign condition. Patients with angina and non-obstructive coronary arteries may have persistent symptoms, repeated healthcare use, impaired quality of life, and increased cardiovascular risk [1-4,8]. This review complements previous aggregate-data syntheses by focusing specifically on INOCA cohorts and by separating comparable from non-comparable estimates rather than combining measures that answer different clinical or statistical questions [3,7-9].

The association is biologically plausible. Endothelial dysfunction, microvascular remodeling, impaired vasodilator reserve, and increased microvascular resistance can produce recurrent ischemia despite the absence of obstructive epicardial disease [2,4-6]. Repeated ischemic injury may contribute to inflammation, myocardial fibrosis, impaired ventricular relaxation, heart failure, and later cardiovascular events [6,14]. Schröder et al. remains central to this evidence base because its large sample size, complete follow-up, and high NOS score provide important narrative support, even though its adjusted estimate was continuous rather than dichotomous [6].

Risk in INOCA may vary by endotype and testing modality. Invasive coronary function testing can distinguish microvascular from vasospastic angina and identify concordant or discordant abnormalities in coronary flow reserve and microvascular resistance [4,9]. Non-invasive tools, including positron-emission tomography, echocardiography, cardiovascular magnetic resonance, and dynamic single-photon-emission computed tomography, provide complementary assessment of myocardial or coronary flow reserve [2,4,6]. Contemporary guidelines support physiology-based evaluation in selected symptomatic patients with non-obstructive coronary arteries [19]. Because the two dichotomous HR studies used invasive assessment, their estimates should not be automatically generalized to all CMD definitions or INOCA phenotypes.

Current evidence does not prove that CMD testing or CMD-directed treatment reduces hard cardiovascular outcomes. Trials guided by invasive coronary function testing improved angina and quality of life but were not powered for MACE, and the WARRIOR trial did not show a significant reduction in MACE among women with suspected INOCA [20-22]. Still, identifying CMD may help with risk stratification, avoiding false reassurance after non-obstructive angiography, guiding symptom-focused therapy, supporting risk-factor control, and planning follow-up [2,19].

Several features support the reliability of this evidence map: prospective protocol registration, duplicate screening and extraction, structured risk-of-bias assessment, explicit handling of overlapping cohorts, and a transparent effect-measure eligibility audit. The main constraints are the observational design of all included studies, possible residual confounding, heterogeneous CMD thresholds and MACE definitions, and the small number of studies reporting adjusted dichotomous HRs. Therefore, the findings support a prognostic association but do not establish causality. The next step is larger multicenter work using harmonized CMD criteria, standardized MACE definitions, clear adjustment sets, and both continuous and dichotomous CMD reporting to clarify whether CMD is only a risk marker or also a modifiable treatment target.

## Conclusions

Available observational evidence suggests that CMD may be associated with increased risk of MACE in patients with ischemia and non-obstructive coronary arteries. Two independent cohorts reported adjusted dichotomous hazard ratios of approximately 3 for a CMD-versus-no-CMD contrast, while the largest and highest-quality cohort supported the prognostic relevance of impaired CFVR using a continuous adjusted estimate. However, heterogeneity in CMD definitions, diagnostic modalities, exposure scales, effect measures, and adjustment strategies prevents reliable quantitative pooling. Larger prospective multicenter studies using standardized CMD definitions, harmonized MACE outcomes, adequate follow-up, and consistently adjusted time-to-event analyses are needed.
